# Facile one-pot green synthesis of Ag–ZnO Nanocomposites using potato peeland their Ag concentration dependent photocatalytic properties

**DOI:** 10.1038/s41598-020-77426-y

**Published:** 2020-11-19

**Authors:** Fahad A. Alharthi, Abdulaziz Ali Alghamdi, Nabil Al-Zaqri, Hamdah S. Alanazi, Amjad Abdullah Alsyahi, Adel El Marghany, Naushad Ahmad

**Affiliations:** 1 Department of Chemistry, College of Science, King Saud University, Riyadh, 11451 Kingdom of Saudi Arabia; 2 Department of Chemistry, Faculty of Science, Suez University, Suez, Egypt

**Keywords:** Materials science, Nanoscience and technology

## Abstract

Herein, a facile green synthesis route was reported for the synthesis of Ag–ZnO nanocomposites using potato residue by simple and cost effective combustion route and investigated the photocatalytic degradation of methylene blue (MB) dye. In the preparation potato extract functioned as a biogenic reducing as well as stabilizing agent for the reduction of Ag + , thus eliminating the need for conventional reducing/stabilizing agents. Ag–ZnO nanocomposites with different Ag mass fractions ranging from 2 to 10% were characterized by using XRD, FT-IR, XPS, SEM, TEM, and UV–Vis spectroscopy. XRD analysis revealed that the as prepared Ag–ZnO nanocomposites possessed high crystallinity with hexagonal wurtzite structure. TEM and SEM images showed that the Ag–ZnO nanocomposites in size ranging from 15 to 25 nm have been obtained, and the particle size was found to increase with the increase in percentage of Ag. FTIR results confirmed the characteristics band of ZnO along with the Ag bands. XPS analysis revealed a pair of doublet with peaks corresponding to Ag and a singlet with peaks corresponding to ZnO. With the increase of concentration of Ag in ZnO, the intensity of NBE emission in the PL spectra was observed to be decrease, resulted to the high photocatalytic activity. Photocatalytic properties of Ag–ZnO nanocomposites evaluated against the MB dye under visible-light irradiation showed superior photodegradation of ~ 96% within 80 min for 2% Ag–ZnO nanocomposites. The apparent reaction rate constant for 2% Ag–ZnO nanocomposites was higher than that of other nanocomposites, which proved to be the best photocatalyst for the maximum degradation of MB. Furthermore, various functional parameters such as dosing, reaction medium, concentration variation were performed on it for better understanding. The enhancement in photocatalytic degradation might be due to the presence of Ag nanoparticles on the surface of ZnO by minimizing the recombination of photo induced charge carriers in the nanocomposites.

## Introduction

Clean and fresh water, which are unevenly distributed on the face of earth, are great need for living beings and our immediate natural environment. Human fluid is significantly made of water (75%); it is essential medium for many biochemical reactions like dissolution of biomolecules proteins, carbohydrates and important activities such as blood transport, digestion, and electrical charged balanced^1^. It provides healthy habitat for aquatic organisms and plants by capturing of balanced amount of sunlight and finally preserve the water ecosystem. Besides human activities, rapid urbanization, climate change, inefficient water management, and world population, the real contributor for the pollution and reduction of available clean water and natural ecosystem are petroleum, pesticides, toxic heavy metal ions, chemicals, greenhouse gases, personal care products wastes and non-degradable dyestuff released from different kind of industries, factories and houses, which are currently become a serious global challenge for the safety of environmental among scientific communities and regulation authorities^2–4^.

Among various wastewater agents, industrial effluents of dyes from plastics, textile, paint, leather and paper industries are frequently released into the water bodies and causes death of living beings and adverse effects on surroundings by accumulation, which have following harmful features: resistant to degrade on exposure to light, chemicals and water, persistent in nature because of their stable chemical structure, carcinogenic aromatically and mutagenic, comparatively higher toxicity, and sunlight opacity due to the its color^5–8^. Therefore, due to the serious impact of dyes, its mineralization or removal before disposal by sophisticated environmental friendly green routes and effective integration of norms as well as environmental policies are seriously very important for conservation of water system, even though it plays a great role in the economic development of any country, widely used science and technology that can make the human life beautiful.

Among various reported techniques for treatment of wastewater^9–11^, photocatalytic process by the application of nano-ranged materials, complete mineralization of the dyes over its surface, is reliable, high sensitive with reasonable costs and environmentally safe technique^12–21^. Briefly in this process, the nanomaterials illuminated by light of the proper wavelength generate active species, which oxidize the organic compounds dissolved in water. Numerable nanomaterials are applied on wastewater treatment but most of them have low catalysis, adsorption capacity, limitations of photocatalytic efficiency, light absorption, and high production costs^22,23^. The current interest for researcher or pollutant management to improve photocatalytic performance either by changing electronic structural properties such as charge transfer, band gap, morphology and particle size or doping of metal and non-metal^23–26^ in advanced dye-sensitizing nanomaterials. Therefore, it is highly needed to design the appropriate electronic structure or doping of the nanomaterials for potential photocatalytic activity.

One of well-known dye sensitive semiconductor is multifunctional zinc oxide nano-oxide which have capability of not only to degradation of complex-stable dye stuff but also very useful for the removal of other hazardous chemicals because of their unique properties such as optical band gap energy (3.37 eV), high-exciton binding energy (60 meV), biocompatibility, high-electron communication features^27,28^. Recently ZnO with various morphologies comprised 0D, 1D, 2D, and 3D^29–32^ as well as dopants^27,33,34^ (metal and non-metal dopants) have been prepared with high photocatalytic efficiency.

The response of ZnO in UV range is well known, while, in order to extend the spectral response of ZnO in the visible range, anchoring of metal particles on the surface of ZnO nanostructures have been studied by various researchers. In a report, Georgekutty et al.^35^ reported the use of non-aqueous method, on the other hand, Chen et al.^36^ deposited Ag on ZnO by using photochemical reaction to prepare Ag/ZnO composite and performed their photocatalytic studies. In another work, Deng et al.^37^ showed that the improvement in charge separation and enhancement in the efficiency of the degradation was ascribed to the Schottky barrier in the regions between Ag and ZnO. It has been shown that the photogenerated electrons from the semiconductor can be trapped in Ag, which allows holes to form hydroxyl radicals that can then react with the organic species, resulting in their degradation. Nevertheless, most of these reported techniques utilize unsafe chemicals, which can cause a serious damage if persist in the environment. Thus, a novel, safe and cost effective method for the preparation of metal − metal oxide nanocomposites needed.

In recent years, green synthesis as a valuable alternatives route to the chemical methods for the preparation of nanoparticles using natural sources including micro-organisms, plants or plant extracts were suggested^38–42^. Among these, the use of plant extracts has shown enormous interest in the synthesis of nanoparticles. More specifically, the potato is tuberous crop which is well known due to its carbohydrate contents where the predominant form of this carbohydrate is starch. Starch, a natural polymer is abundant, renewable, inexpensive and widely available and could be efficiently used as templates for nanoparticles growth^43,44^.

Keeping above facts in mind, in this work, a facile, green, low-cost and one-pot method for synthesis of Ag–ZnO nanocomposites using potato peel without any additives such as reducing agent, acids and organic solvents were reported. The photocatalytic properties of these Ag–ZnO nanocomposites containing various concentration (1%, 2% 8%, 10%) of Ag in ZnO were investigated by measuring the degradation of MB under visible light irradiation. Various parameters such as effect of Ag concentration, catalyst dosage, MB dye concentrations, and pH on dye degradation were also presented. Based on the obtained results and their discussions, a possible mechanism related to the degradation of MB dye with Ag–ZnO nanocomposites has been discussed investigated in detail. The experimental results showed that the as-obtained ZnO nanorods with 2% Ag in ZnO exhibited excellent photocatalytic activity than other nanocomposites. This study will provide the platform to synthesize ZnO based nanocomposites by route using potato or other biomaterial by green method, and the photocatalytic properties could be easily tuned for future applications. We believe that this simple and one-step method can suitably be scaled up for large-scale synthesis.

## Materials and methods

The chemicals such as zinc nitrate hexahydrate (Zn(NO_3_)_2_·6H_2_O), silver Nitrate (AgNO_3_), Methylene Blue dye (MB), were purchased from Sigma-Aldrich-Germany and used as received. The glassware was used with analytical boro-silicate grade.

### Experimental details

#### Synthesis of Ag–ZnO nanocomposites using potato peels

Fine powder of potato peels (0.594 g) was mixed with 0.291 g Zn(NO_3_)_2_·6H_2_O (99.999%, Sigma-Aldrich-Germany) and 1 wt% AgNO_3_ (99.999%, Sigma-Aldrich-Germany) had been taken in borosil glass container. After adding 10 mL deionized water, the solution was stirred for 10 min on a hot plate at 70 °C. The resulting solution was centrifuged, washed with DI water and dried at 50 °C. The as-prepared product was further heated in air in a muffle furnace at 500 °C for 10 min to obtain Ag–ZnO nanocomposites. The obtained product was used for the characterizations. Similarly, other compositions namely 2% Ag–ZnO nanocomposites, 8% Ag–ZnO nanocomposites, and 10% Ag–ZnO nanocomposites were prepared and characterized in terms of their morphological, structural and optical properties, and were used as photocatalysts for the mineralization of toxic MB dye.

### Characterization

The crystallinity of fine powders was recorded through X-ray diffractometer (Rigaku Ultimate 1V, Japan) with Cu_Kα_ radiation source (λ = 1.5417 Å) in 2*θ* range of 10–80°. The Fourier transform infrared spectroscopy (FTIR, Bruker Vertex 70, Germany) measurements were conducted from the range of 4000–400 cm^−1^ with KBr pellet method at room temperature. The morphological aspects and particle size of fabricated catalysts were examined with scanning electron microscope (SEM) (JEOL JSM7600F, Japan) and transmission electron microscope (TEM, JEOL-21000F, Japan). The chemical states and surface element composition were determined by X-ray photoelectron (XPS) in omicron with a monochromatic Al_Kα_ radiation source and charge neutralizer. The C 1s line was taken as an internal standard at 284.6 eV. Both wide-range survey spectra and detailed spectra (for Ag 3d, Zn 2p, O 1s and C 1s) were collected at 300 W.

### Photocatalytic activity measurements

For the photocatalytic properties of Ag–ZnO nanocomposites, MB was chosen as water pollutant modal. Degradation of MB by Ag–ZnO nanocomposites were carried out in a photocatalytic reactor, which consist of a 250 W visible lamp and 37 cm long quartz tube of 100 ml capacity. Aqueous solution (5 ppm) of the MB and fabricated catalyst (10 mg) was photocatalyzed in a quartz reactor at room temperature under the UV light irradiation. The prepared suspension was sonicated in the dark for the dispersion and establishment of adsorption–desorption between the MB and the catalyst before irradiation. After that 2 ml centrifuged solution was taken out from reactor and its absorbance was recorded at UV–Vis spectrophotometer by monitoring 664 nm wavelength in the range of 200–800 nm for 80 min. A MB solution in absence of any photocatalyst was used as control.

## Results and discussions

### X-ray diffraction

**Figure 1:**
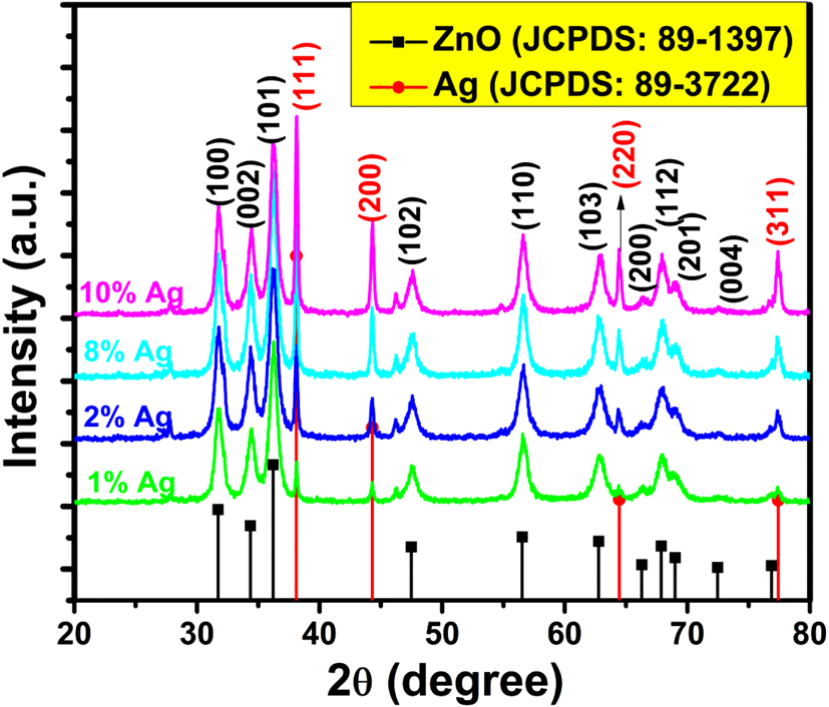
XRD patterns for Ag–ZnO nanocomposites (**a**) 1%, (**b**) 2%, (**c**) 8%, (**d**) 10% Ag.

Figure 1 shows the XRD patterns of Ag–ZnO nanocomposites in which two different sets of XRD pattern were observed associated with Ag and ZnO. The typical hexagonal wurtzite structure with P6_3_mcsymmetry of ZnO NPs showed peaks positioned at 2*θ* values of 31.64°, 34.45°, 36.23°, 47.61°, 56.62°, 62.97°, 66.45°, 67.85° and 68.97°, which are indexed as (100), (002), (101), (102), (110), (103), (200), (112) and (201) planes, respectively, and well matched with the JCPDS, File No. 036-1451^45^. In addition, the presence of the four specific peaks at 38.15°, 44.36°, 64.53° and 77.39° clearly matched with (111), (200), (220) and (311) planes of silver (Ag), respectively. All the peaks in XRD pattern can be readily indexed to a face-centered cubic structure of Agas per available literature (JCPDS, File No. 4-0783)^46^, confirmed the presence of Ag in the sample. The peak intensity of Ag phase for Ag–ZnO nanocomposites was intensified and sharper with the increment of Ag contents, which suggest that Ag metallic phase has been successfully formed on the surface of ZnO-NPs rather than incorporation into the ZnO lattice. This could be due to the fact that the ionic radius of Ag + (126 pm) is larger than that of Zn^2+^(74 pm), which resulted to the formation of metallic Ag, and no shift in the peak positions of Ag–ZnO nanocomposites indicates that Ag particles are positioned on the surfaces of well crystalline ZnO-NPs^47^. The crystallite size of the Ag–ZnO nanocomposites calculated using the Scherrer formula^45^ was found to increase from 12 to 20 nm with the increase of Ag concentration in ZnO. This increase in crystallite size ascribed to the Ag nanoparticles anchoring on the surface of ZnO.

### Fourier transform infrared (FTIR) spectroscopy

**Figure 2:**
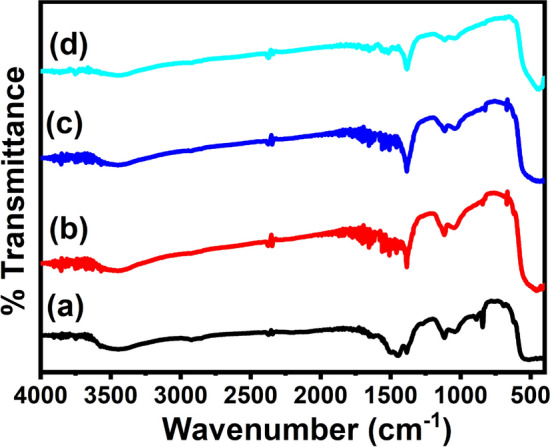
FTIR spectra Ag–ZnO nanocomposites (**a**) 1%, (**b**) 2% (**c**) 8%, and (**d**) 10% Ag.

The chemical composition in terms of functional groups of the prepared products was analyzed by the FTIR spectroscopy in the range of 400–4000 cm^−1^ and shown in Fig. 2. A broad band at 3200–3435 cm^−1^ can be attributed to stretching H-bound water, and 1410–1622 cm^−1^ can be ascribed to the bending vibrations of the hydroxide (–OH) groups absorbed at the surface of samples^48^. The weak bands observed at 2364, 1095 and 1041 cm^−1^ were related to the vibrations of the organic residuals. The sharpest and dominated absorption band was appeared at 530 cm^−1^ which might be correlated to the M–O bonds (M = Zn and Ag)^49^. No notable shift in the absorption peaks were observed with increasing the Ag concentration but bands became sharpen.

### X-ray photoelectron spectroscopy (XPS) analysis

**Figure 3:**
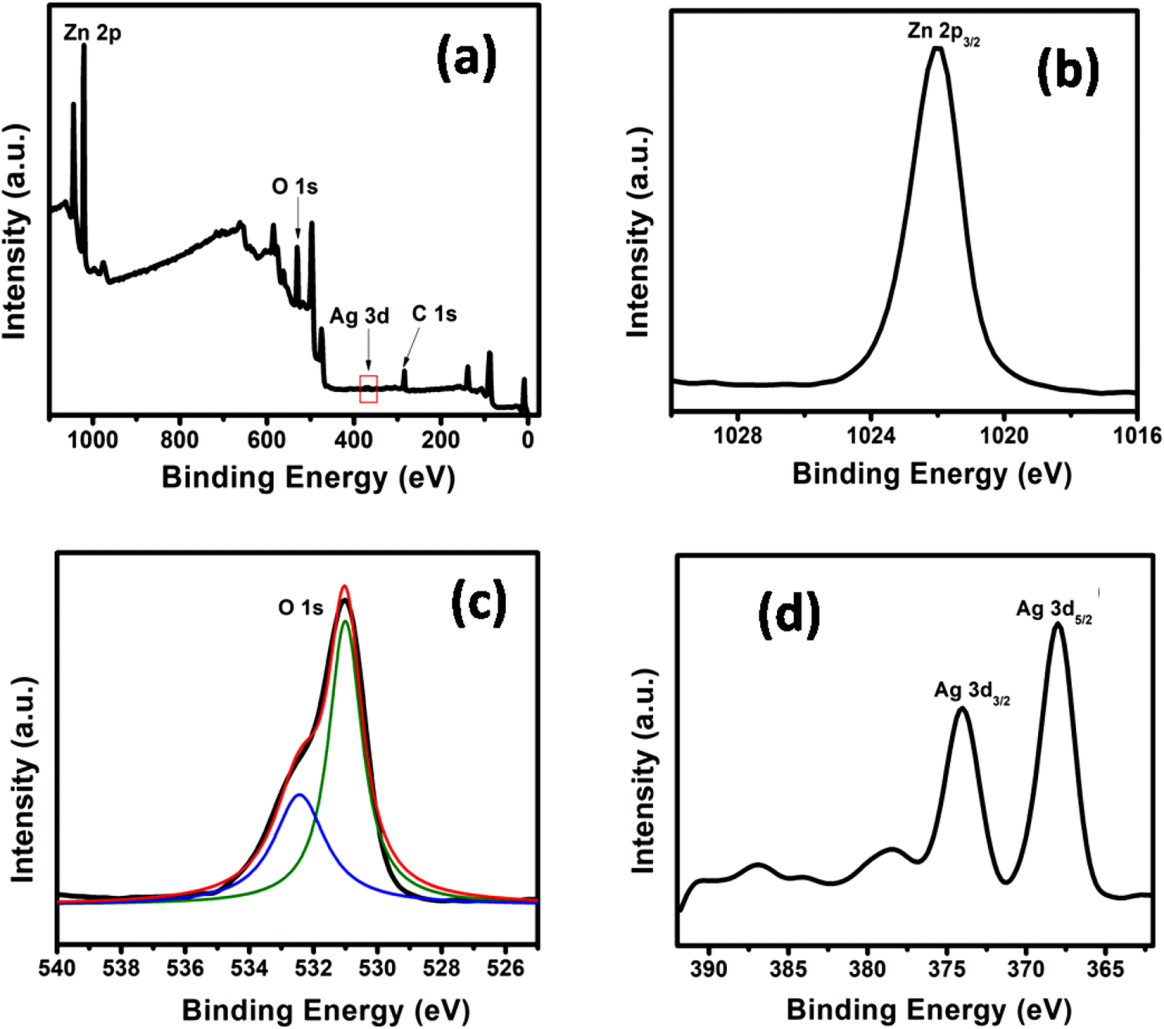
XPS spectra of 2% Ag–ZnO nanocomposites (**a**) survey, (**b**) Zn2p_3/2_, (**c**) O1s, and (**d**) Ag3d spectra.

The XPS technique was used to investigate the chemical states and surface composition of 2% Ag–ZnO-NPs due to its importance in photocatalytic activity. The survey spectra presented in Fig. 3a confirms that Ag–ZnO nanocomposites contains the elemental signals from carbon (C), oxygen (O), Zinc (Zn) and silver (Ag) atoms in the sample, which is consisted with the XRD results. No other impurity elements were observed, which further confirms the high purity of the nanocomposites. The appearance of carbon peak (not shown) (C 1s = 284.8 eV) was mainly related to the residual carbon from the sample as well as the hydrocarbons from the XPS instrument^50^. High resolution spectrum of Zn 2p, O 1s, and Ag 3d is shown in Fig. 3b–d, where; the binding energies of Ag–ZnO nanocomposites are slightly different, revealing a strong interaction between Ag and ZnO nanoparticles. As observed in Fig. 3b, there was one peak centered at 1022.06 eV corresponded to the Zn 2p_3/2_, indicating a normal state of Zn^2+^ in the 2% Ag–ZnO nanocomposites. Other peak was centered at 1044.4 eV correspond to the binding energy of Zn 2p_1/2_ (see Fig. 3a). O 1s core-level spectrum (see Fig. 3c) showed two sub-peaks at 532.43 eV (O_I_) and 530.95 eV (O_II_). In the XPS spectrum, the peak at high binding energy assigned as O_I_ is related with the adsorbed oxygen or hydroxide, however, the peak positioned at lower energy assigned as O_II_ peak could be attributed to the lattice oxygen of Ag–ZnO nanocomposites. During photocatalyst is, surface hydroxyl group tends to plays a major role^51^. Figure 3d shows the high resolution spectrum of Ag 3d deconvoluted into three peaks. The peaks positioned at 374.6 eV and 368.6 eV could be related to the Ag 3d_3/2_ and Ag 3d_5/2_ characteristics for metallic silver (Ag°), respectively^52^, confirmed the successfully reduction of Ag ions to produce metallic silver in Ag–ZnO nanocomposites.

### Morphological study

**Figure 4:**
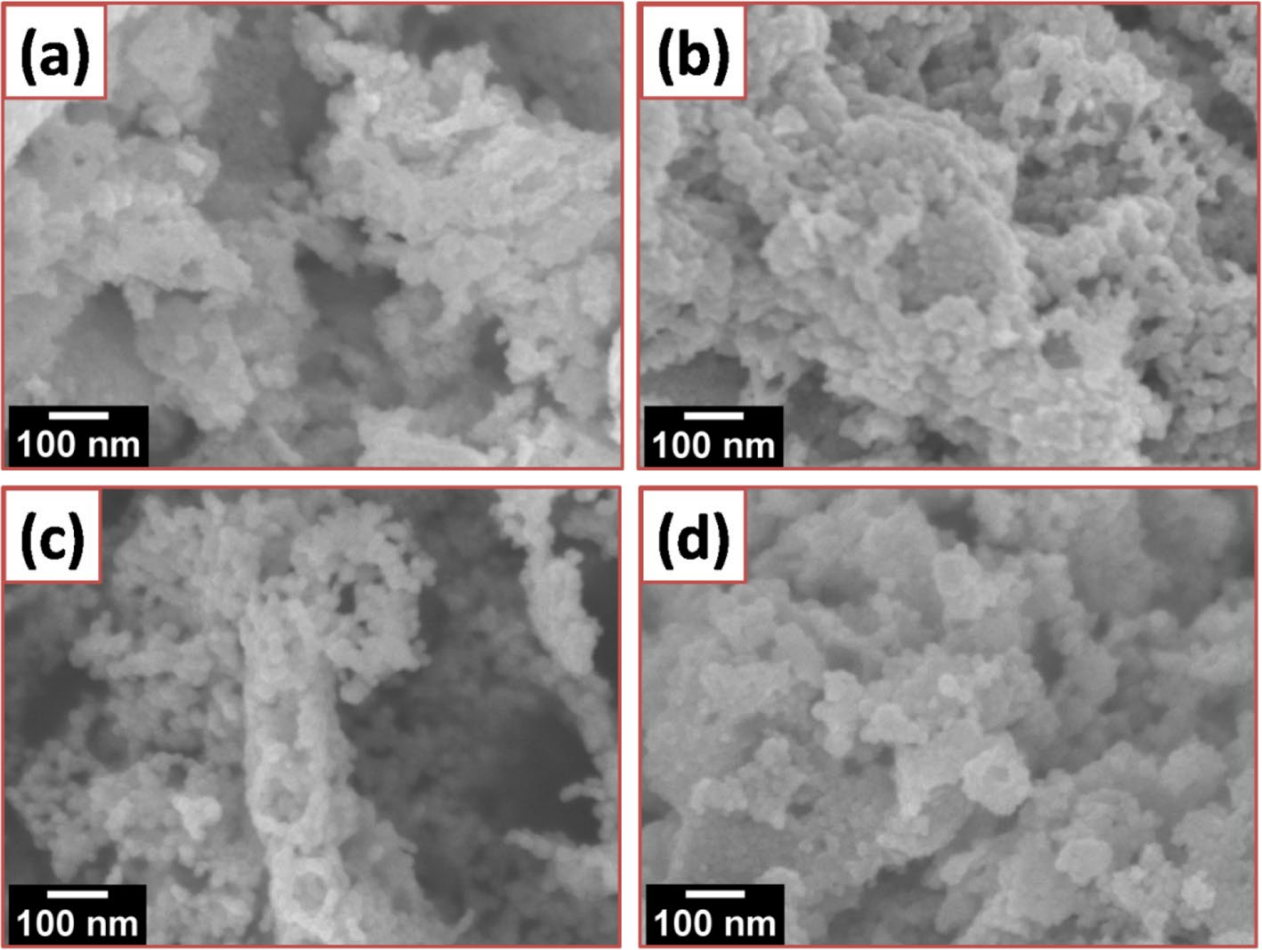
SEM images of different Ag–ZnO nanocomposites (**a**) 1%, (**b**) 2%, (**c**) 8%, and (**d**) 10% Ag.

**Figure 5:**
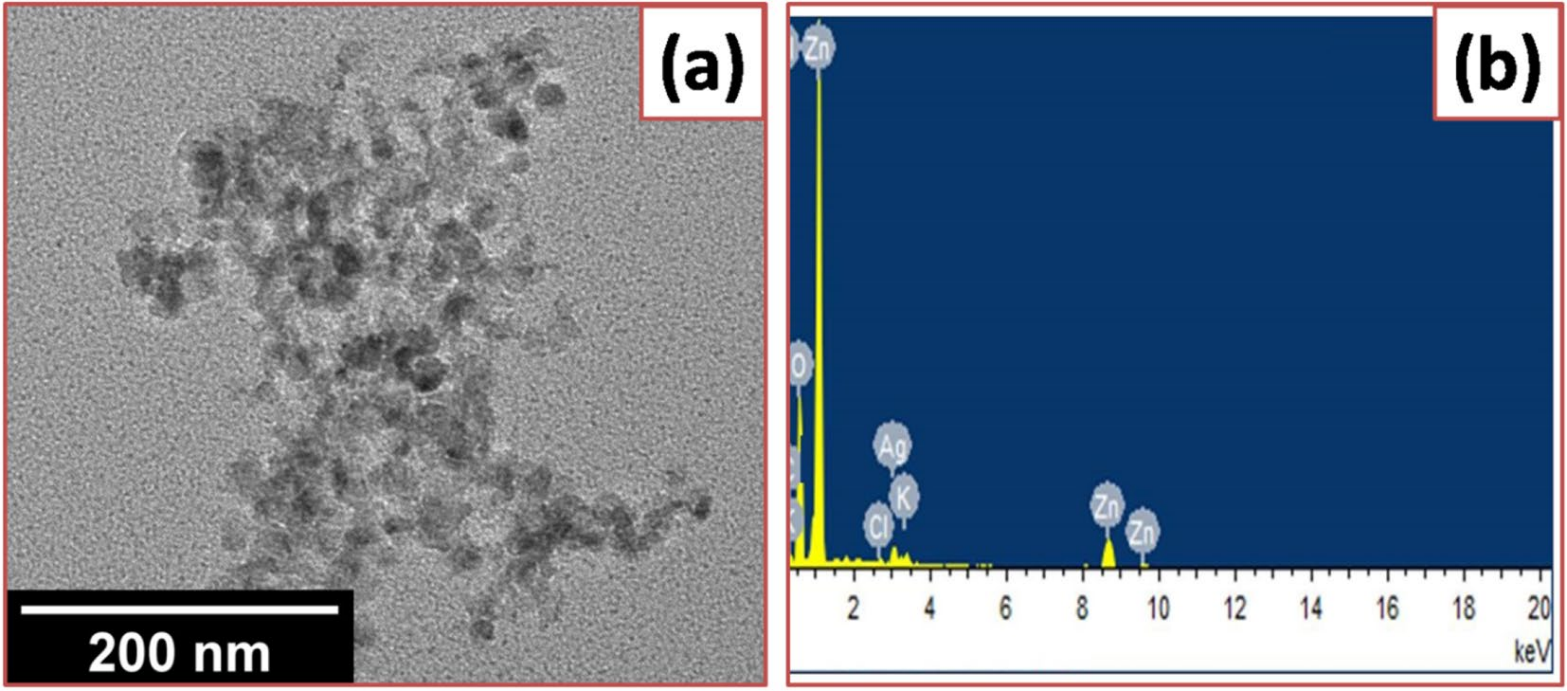
**(a)** TEM images of 2% Ag–ZnO nanocomposites, and (**b**) corresponding EDX spectrum.

**Figure 6:**
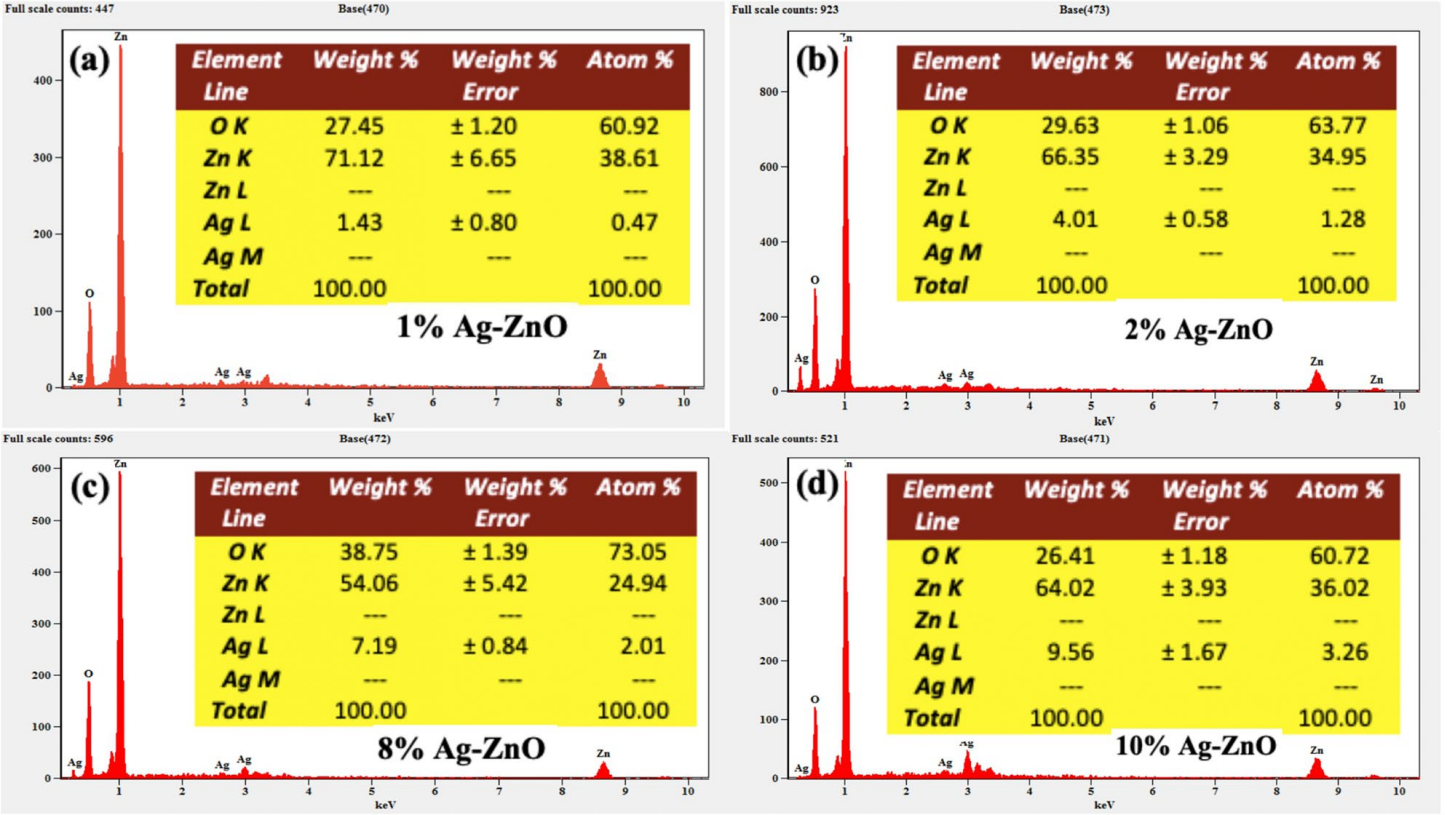
EDX spectra of (**a**) 1%, (**b**) 2%, (**c**) 8%, and (**d**) 10% Ag–ZnO nanocomposites.

The SEM images of various composition are shown in Fig. 4a–d confirmed the co-existence of Ag and ZnO NPs in Ag–ZnO nanocomposites with a size ranging from 15 to 25 nm. It also shows that the ZnO has a low density, and a loose material that is favorable for a catalytic application. It is observed that Ag NPs are very small in size at low concentration but lot of Ag aggregated appeared at surface of dispersed and porous ZnO flower like microforest structures observed, when the amount was increased. To further validate the existence of Ag NPs, the TEM analysis was performed on the more photocatalytic active sample, 2% Ag–ZnO NPs (Fig. 5a), clearly identify a lot of Ag NPs of small and spherical size (7–12 nm) deposited on ZnO surface. This is probably due to the inhibition of Ag doping towards the crystallization and crystal growth of ZnONPs^53^. It can be seen that Ag nanoparticles are anchored on the surface of ZnO, and well distributed over the surface. Figure 5b shows the EDX spectrum of 2% Ag–ZnO nanocomposites in which Zn, O, and Ag signals were detected, which reveals that nanocomposites contain well distributed Ag in ZnO. In order to further determine the Ag content in ZnO with various Ag concentrations, EDX analysis of Ag–ZnO nanocomposites with different Ag content was performed and shown in Fig. 6. It is clear from Fig. 6 and the elemental table (insets) that the Ag content (1%, 2%, 8%, and 10%) in ZnO is well matched with elemental analysis, which indicated that Ag has been successfully incorporated in ZnO.

### Optical properties (PL)

**Figure 7:**
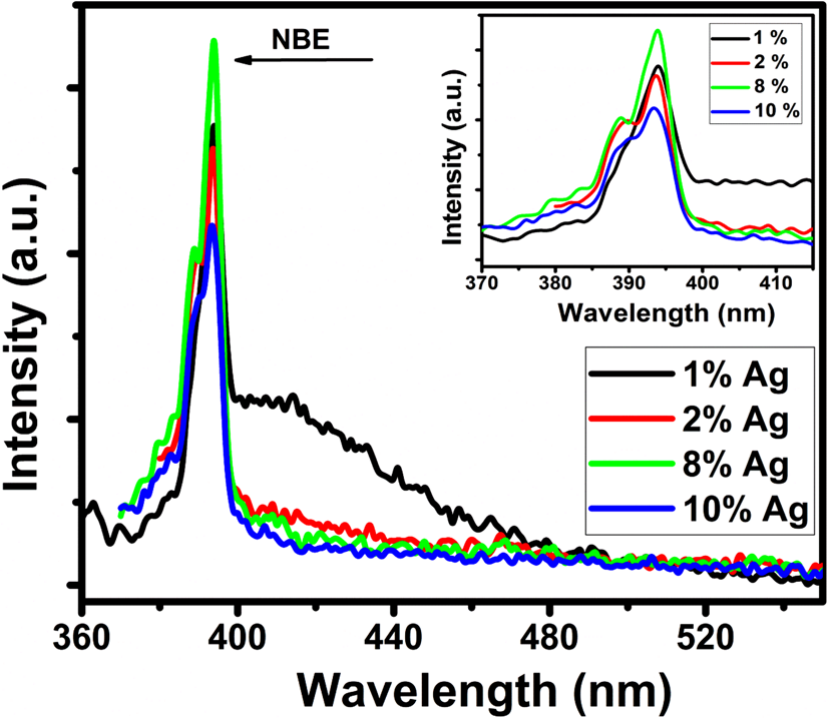
Room temperature PL spectra of Ag–ZnO nanocomposites.

In order to study the charge recombination and migration efficiency of Ag–ZnO nanocomposites, optical properties of Ag–ZnO nanocomposites were evaluated using PL technique as the photocatalytic properties are strongly dependent on the PL intensity and the recombination rate of photogenerated charge carriers. Figure 7 shows the room-temperature PL spectra of the Ag–ZnO nanocompositeswith various concentration of Ag in ZnO. A near-band-edge (NBE) emission at ~ 393 nm was observed and the intensity of NBE emission was found to decrease with the increase of Ag concentration in ZnO, which suggests that the anchoring of Ag NPs could quench the fluorescence from the ZnO nanoparticles and prolong electron–hole pair lifetime^45,54^.

The recombination of free excitons of ZnO could be well explained and related with the NBE emission^55^. Usually, the photogenerated charge carriers separation is directly associated with NBE peaks. For higher recombination rate, the NBE peaks are stronger, which resulted to slower photocatalytic activity^54^. In this work, 2% Ag–ZnO nanocomposites showed a decrease in the NBE intensity, hence, a higher separation rate and lower recombination rate of photo induced charge carriers, which leads to higher photocatalytic activity. While for 8% Ag–ZnO nanocomposites, the intensity was found to increase, showed the lower photocatalytic activity.

### Photocatalytic degradation of MB

**Figure 8:**
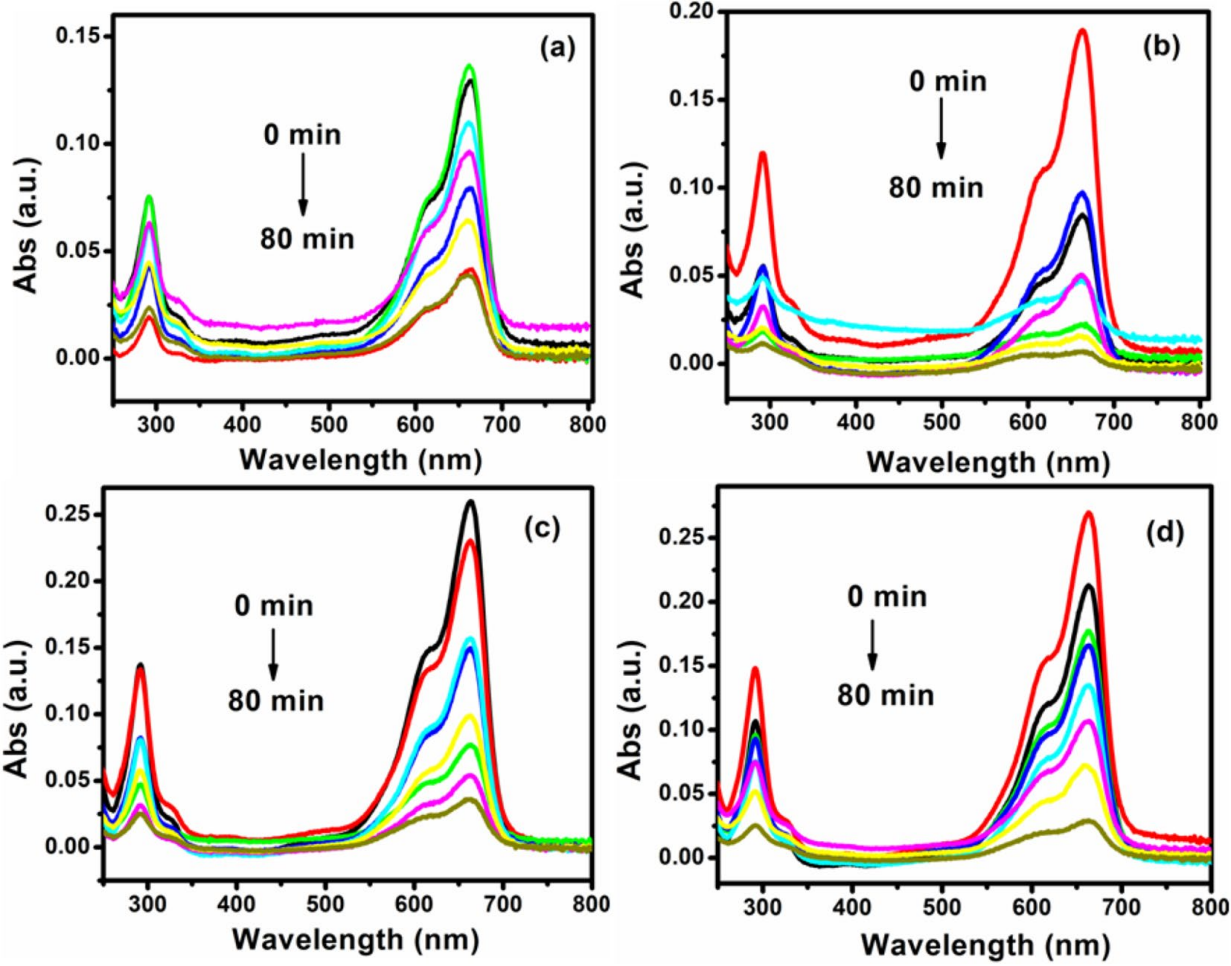
UV–visible absorption spectra of photodegradation of MB in the presence of (**a**) 1%, (**b**) 2%, (**c**) 8%, and (**d**) 10%Ag–ZnO nanocomposites.

Photocatalytic properties of Ag-ZnO nanocomposites with various concentration of Ag in ZnO were studied by decomposition of MB. To study the photodegradation of MB, the change of absorbance at 664 nm wavelength at different time interval was monitored^56^. Figure 8a–d depicts the time-dependent absorption spectra of MB aqueous solutions under visible light irradiation with Ag–ZnO nanocomposites. It is clear from the Fig. 8a–d that in the presence of Ag–ZnO nanocomposites, the maximum absorption of the MB solution was found to decrease with illumination time and disappeared almost completely (~ 96%) after irradiation for about 80 min for 2% Ag–ZnO nanocomposites.

**Figure 9:**
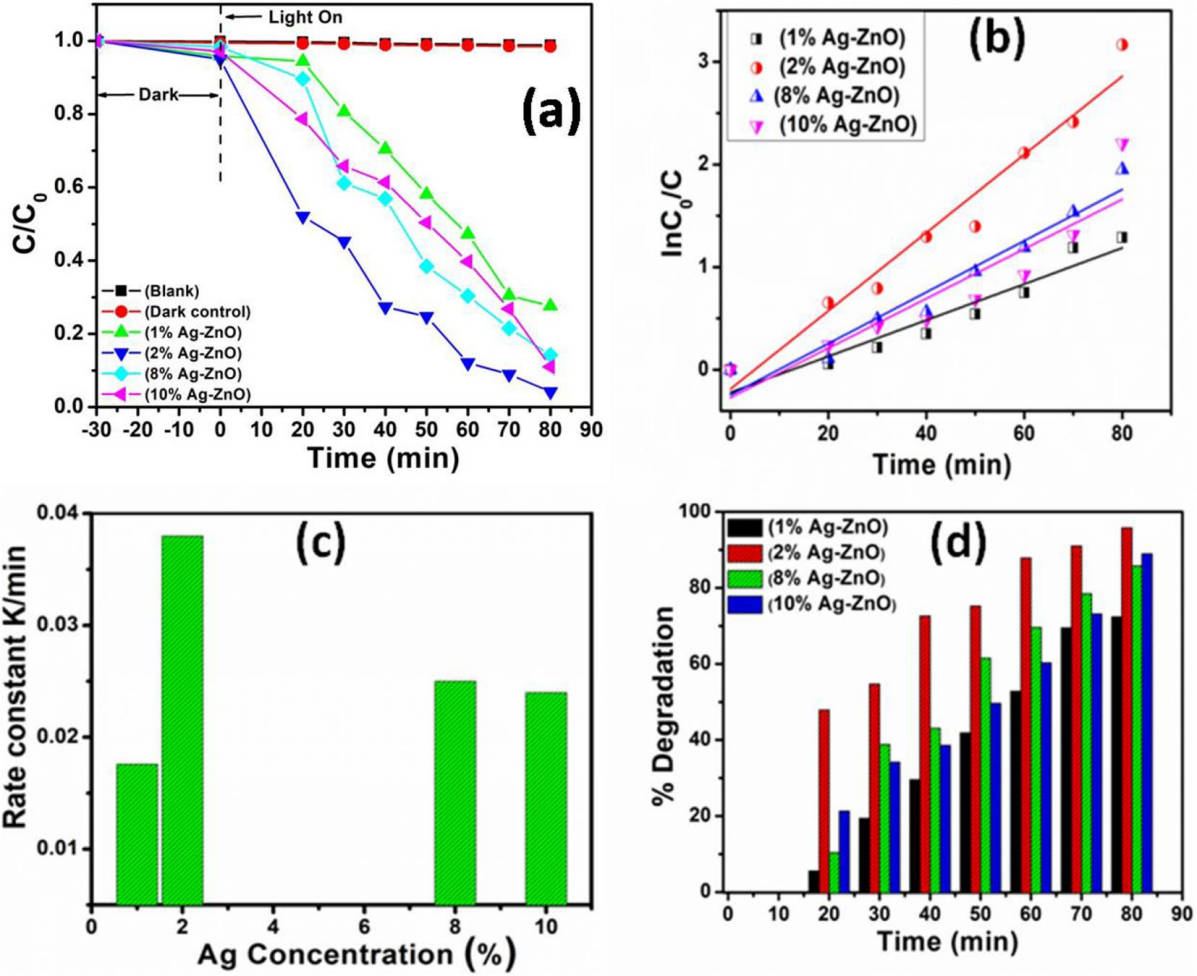
(**a**) C/C_0_ vs time (min) plot for the photodegradation of MB, (**b**) Kinetic relationship of ln(C_0_/C) vs. irradiation time, (**c**) Plot of rate constant vs. Ag concentration, and (**d**) Percentage degradation of MB dye as a function of Ag concentration.

To further evaluate the relationship between photocatalysis and the concentration of Ag in ZnO, studies on relative concentration (C/C_0_) of MB were performed. Figure 9a shows the relative concentration (C/C_0_) of MB as a function of irradiation time for Ag–ZnO nanocomposites, where C corresponds to the concentration of MB at the irradiation time (t) and C_0_ is the concentration of the dye before irradiation. When the suspensions were magnetically stirred in the dark for 30 min to ensure establishment of an adsorption/desorption equilibrium of MB on the sample surface, only slight decrease in the MB solution concentration was observed, which demonstrates that the adsorption of MB on the samples is limited after the adsorption–desorption equilibrium is reached. A control experiment revealed negligible decolorization of the dye solution treated with dye solution with photocatalyst in the dark. The extent of decolorization was similar to the blank sample comprising dye solution illuminated with UV light, without photocatalyst. It could be clearly seen from Fig. 9a that for all Ag–ZnO nanocomposites placed in the solution of MB, the concentration of MB solution was decreased with irradiation time, which indicates that all the Ag–ZnO nanocomposites show UV-light photocatalytic properties for the degradation of MB. Particularly, the photocatalytic activity of the 2% Ag–ZnO nanocomposites showed excellent activity of degradation for 80 min of irradiation ac compared with 1%, 8%, and 10% Ag–ZnO nanocomposites.

To obtain the reaction rate of the Ag–ZnO nanocomposites photocatalysts to understand the role of Ag concentration in ZnO, the kinetic behaviour of these photocatalysts was further studied and the results obtained were shown in Fig. 9b. Generally, the value of ln(C_0_/C) and the irradiation time follows linear behaviour^45^. The photocatalytic process can be regarded as pseudo-first-order reaction and the rate equation is expressed as ln(C_0_/C) = kt, where, t is reaction time, k is the apparent reaction rate constant, and C_0_ and C are the concentration of MB at time of 0 and t, respectively. It is found that the apparent reaction rate constant K for the degradation of MB was found to be 1.76 × 10^–2^ min^−1^, 3.80 × 10^–2^ min^−1^, 2.49 × 10^–2^ min^−1^ and 2.39 × 10^–2^ min^−1^, respectively for 1% Ag–ZnO nanocomposites, 2%, 8%, and 10% Ag–ZnO nanocomposites, respectively. Figure 9c shows the relationship between the reaction rate k and various concentration of Ag in ZnO. It is clear from Fig. 9c that the reaction rate constant for 2% Ag–ZnO nanocompositesis higher than that of other nanocomposites, which reveals the higher photocatalytic activities of 2% Ag–ZnO nanocomposites.

In order to get more insight on the photodegradation with variable concentration of Ag in ZnO, the percentage (%) degradation of MB dye as a function of Ag concentration was calculated and shown in Fig. 9d. It is clear from Fig. 9d that the % degradation increases with the increase in concentration of Ag. The nanocomposites containing 2% Ag in ZnO showed the higher photocatalytic performances of ~ 96% within 80 min of irradiation, while only 72%, 85%, and 89% degradation efficiency of MB was observed for 1%, 8%, and 10% Ag–ZnO nanocomposites, respectively. This clearly indicates that 2% Ag–ZnO nanocomposites based photocatalyst is more superior to than that of others. These results are in close agreement with the studies observed from PL NBE emission.

**Figure 10:**
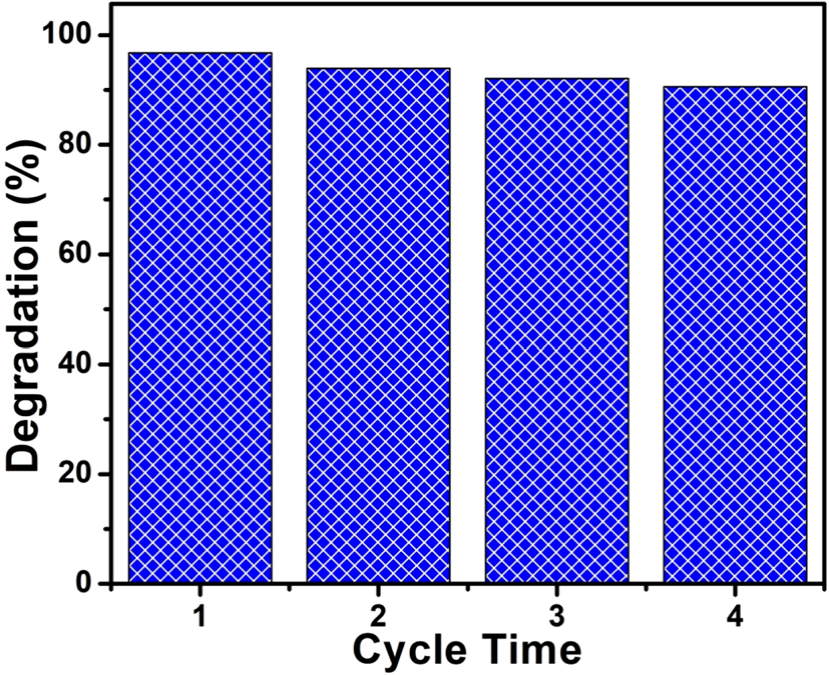
The stability and reusability of 2% Ag–ZnO nanocomposites for photodegradation of MB.

In order to estimate the photocatalytic stability of 2% Ag–ZnO nanocomposites photocatalysts, the time courses of photocatalytic degradation of MB were performed as shown in Fig. 10. Slight decrease of 6% in degradation of MB was observed in repeated runs for the photocatalytic reaction of 80 min, which reveals that 2% Ag–ZnO nanocomposites have good stability and reusability performance and can be a potential candidate for practical photocatalysis applications.

#### Effect of silver doping

**Figure 11:**
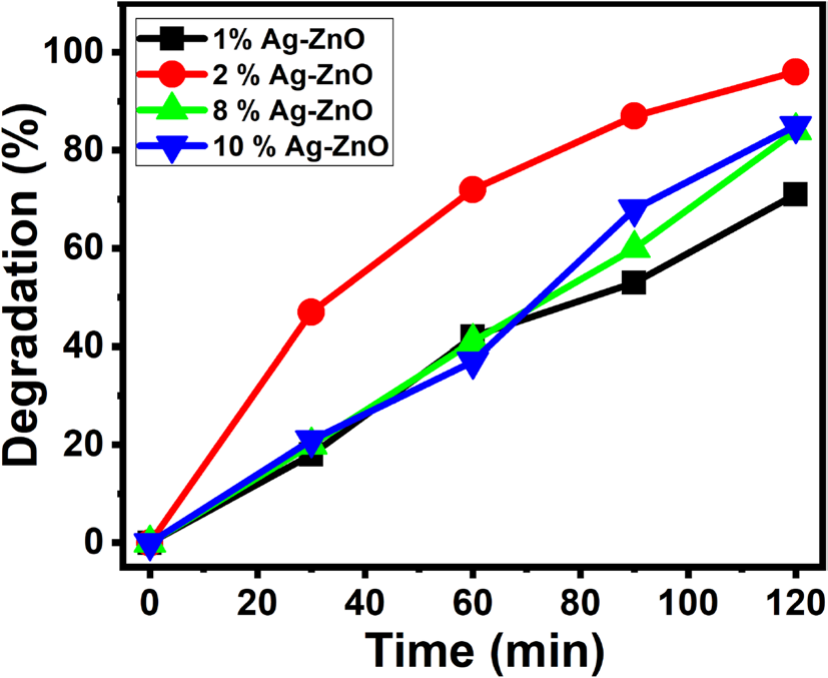
Effect of Ag concentration on photocatalytic degradation of MB.

About 100 mL of 5 ppm MB solution was mixed with 10 mg of catalyst and exposed to visible light. Figure 11 shows the degradation of MB at different concentrations of Ag–ZnO NPs (1, 2, 8 and 10 Mol%). The maximum degradation efficiency was obtained for 2% Ag–ZnO NPs (~ 95% for 120 min) because of the uniform dispersion of Ag on the surface ZnO NPs, which enables the separation and migration of charge carriers^57^. Furthermore, the optimum load of Ag concentration was performed for the better degradation rates of MB. At low Ag concentration, lower degradation was noticed, due to the less accessibility of Ag for electrons absorption by the conduction band (CB) of ZnO. Agglomeration of the Ag cluster leads to the blocking of nanocrystals at the higher concentration of Ag loading more than 2%, which results in the lowering of photocatalytic MB degradation^58^. From the outcomes, it is found that the activity is less when Ag loading is more than or less than 2%. The order of increasing photocatalytic activity for dye degradation as follows, 1% < 8% < 10% < 2% of Ag concentration.

#### Effect of catalyst dosage

**Figure 12:**
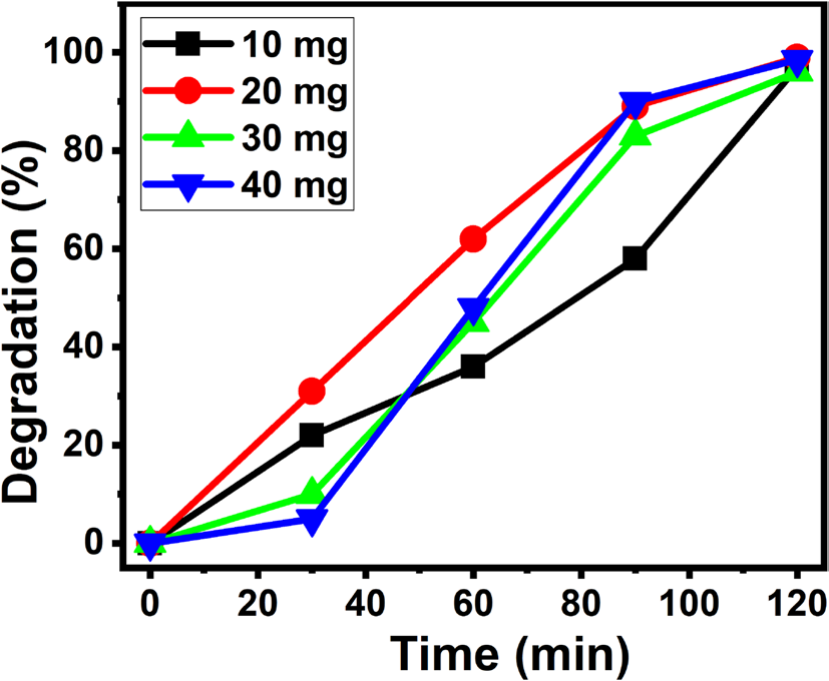
Effect of catalyst dosage on the rate of MB degradation.

In order to know the optimal catalytic dosage for the efficient degradation of MB, experiment was carried out at different weights (10, 20, 30 and 40 mg) of 2% Ag–ZnO NPs by keeping the other parameters constant (100 mL of 5 ppm MB dye) and the results were shown in the Fig. 12. It was observed that the rate of degradation increases, as the concentration of the catalyst load increases from 10 to 20 mg/100 mL, this increases the active sites on the surface of the catalyst, which enhances the absorption of number of MB dye. As we further increase the catalyst dosage from 20 to 30 mg/100 mL, there is a lower degradation of MB. The fact may be due to the blocking and hindrance of light through the solution on the surface of the catalyst^59^. In addition to that high-level concentration of the photocatalytic powder makes the particle aggregation which significantly reduces the active sites on the catalyst surface and hence reduces the efficiency of photocatalytic degradation.

#### Effect of MB dye concentration

**Figure 13:**
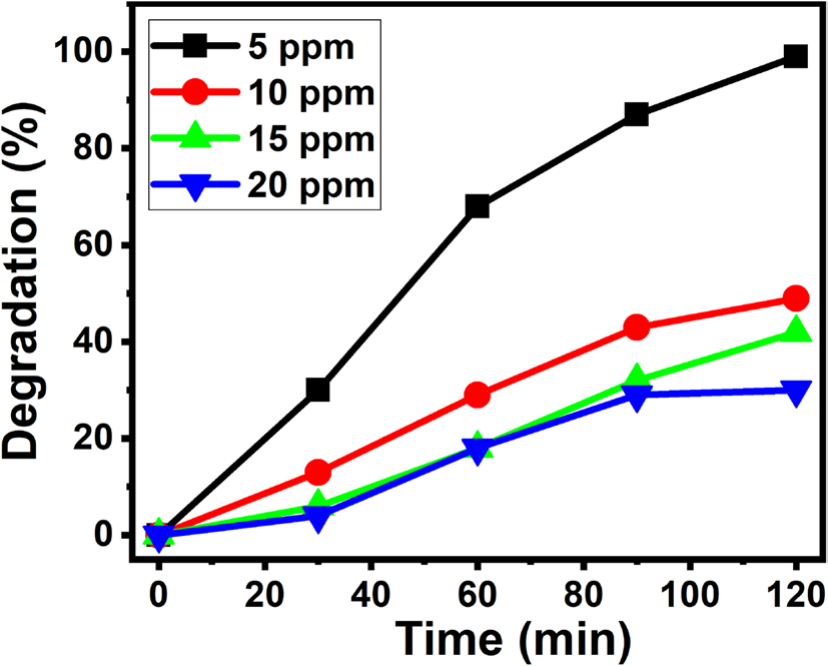
Effect of MB dye concentration on the rate of degradation.

Under the examination of photocatalytic MB degradation, its concentration is very significant parameter. So that, the evaluation of optimal concentration for the efficient oxidation is identified under the set of constant catalytic load (20 mg/100 mL) and varying the dye amount from 5, 10, 15 and 20 ppm under the influence of visible irradiation. From Fig. 13, it is observed that as the concentration increases from 5 to 20 ppm, the rate of degradation decreases. This may be due to the lowering of light penetrating power, when the dye concentration is enhanced, which lowers the production of photogenerated-electrons and holes ultimately leads to lowering the rate of photodegradation^60^.

#### Effect of pH on dye degradation

**Figure 14:**
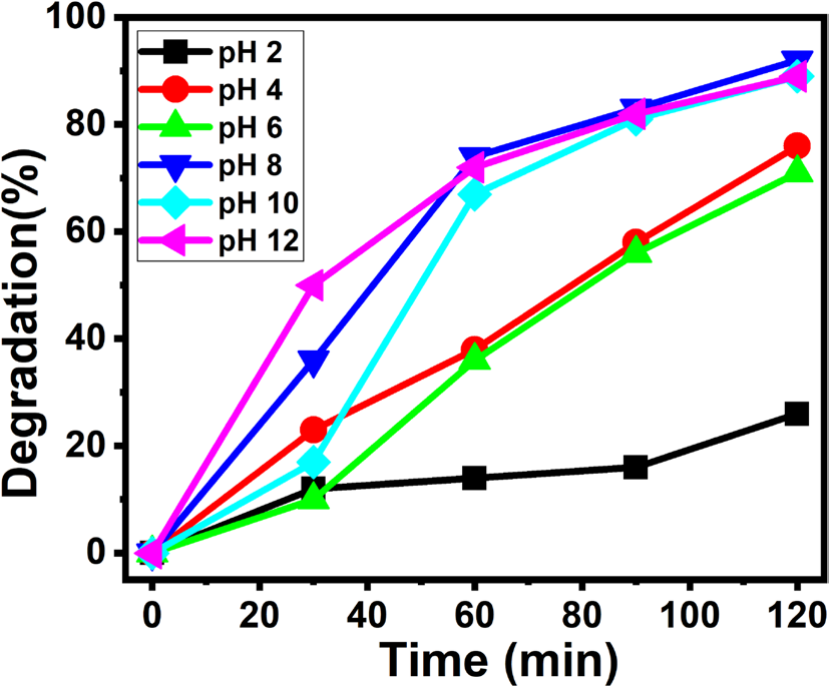
Effect of pH on the rate of degradation.

The study of effect of pH on the degradation of MB dye using 2% Ag–ZnO NPs are shown in Fig. 14. The highest degradation efficiency of MB shown under basic condition (pH 8, 10, 12) and lower activity was observed under acidic condition. This study was confirmed that the MB showing more degradation in the alkaline condition specifically at pH 8 and this was due to the adsorption of more number of MB dyes on selected catalyst. In presence of acidic medium, MB dye shows least degradation, which is due to the dissolution of Ag–ZnO derivatives and forms the corresponding salts^61^.

The increase in the rate of degradation of MB dye under basic medium is mainly due to the formation of more number of •OH from the ^–^OH rather than H_2_O. Hence, the rate of degradation of MB is more. Further, adsorption of more number of MB onto catalyst in basic pH caused by the reduction and oxidation between the MB dye and catalyst. Hence, the photocatalytic dye degradation is low at acidic pH compared to basic pH and similar results of photocatalytic degradation were found for ZnO analogues ^62^.

1 $$ {\text{h}}_{{{\text{VB}} + }} + {\text{OH}}^{ - } \to {\text{OH}} \bullet $$

2 $$ {\text{MB}} + {\text{ h}}_{{{\text{VB}} + }} \to {\text{oxidation}}\;{\text{products}} $$

3 $$ {\text{MB }} + {\text{ e}}_{{{\text{CB}} - }} \to {\text{reduction}}\;{\text{products}} $$

#### Mechanism of degradation of MB using 2%Ag–ZnO NPs

**Figure 15:**
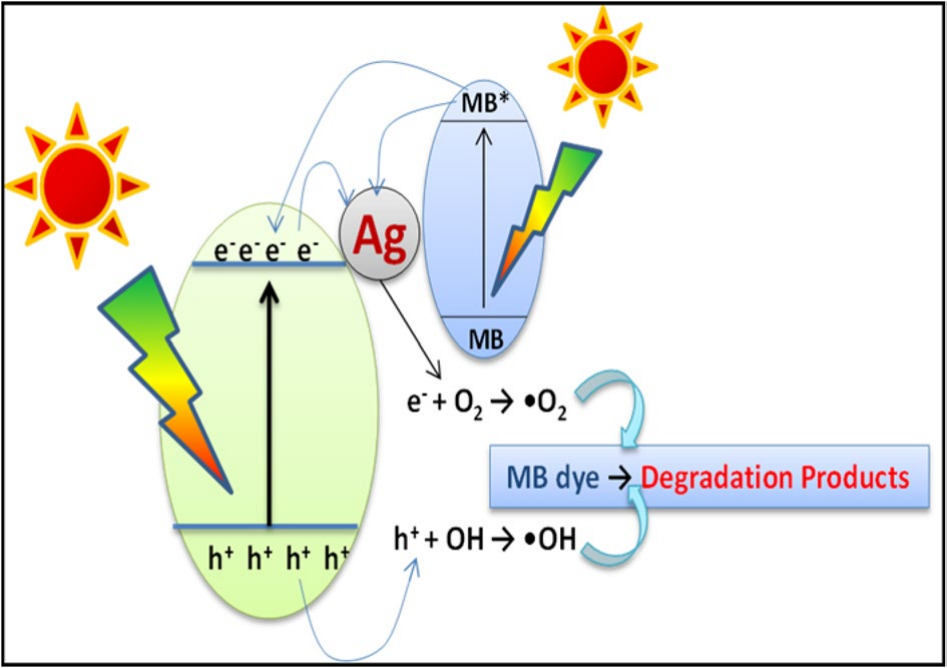
Schematic representation of MB dye degradation by Ag–ZnO Photocatalyst.

When the light of certain wavelength (which has equal to or higher than the wavelength of band gap of the catalyst) absorbed byAg–ZnO nanocomposites, there will be jumping of electrons from valance band (VB) to conduction band (CB). This creates the holes at the VB, which are electron deficient species. At the same time, the electrons are more gathered at the CB, which are electron rich species. The electrons at the CB reacts with O_2_ to form •O_2_^−^ and holes at the VB reacts with water to form •OH. These created intermediates are highly reactive as well as strong oxidizing in nature and oxidizes the MB dye into to CO_2_, H_2_O and corresponding mineral acids as degradation products. These results recommend that Ag NPs anchored on the surface of ZnO might help in increasing the formation rate of •O_2_^−^ and •OH reactive species, and concurrently help the degradation of organic pollutants. A probable mechanism of MB degradation by Ag–ZnO is shown in below scheme, and graphically represented in Fig. 15.

4 $$  {\text{Ag}}{-}{\text{ZnO}} + {\text{h}}\nu  \to {\text{Ag}} - {\text{ZnO }}\left( {{\text{h}}^{ + } _{{{\text{vb}}}}  + {\text{ e}}^{ - } _{{{\text{cb}}}} } \right)  $$

5 $$ {\text{OH}} -_{{{\text{ads}}}} + {\text{ h}} +_{{{\text{vb}}}} \to {\text{OH}}\cdot_{{{\text{ads}}}} \left( {{\text{in}}\;{\text{basic}}\;{\text{medium}}} \right) $$

6 $$ {\text{MB}} + {\text{OH}}\cdot_{{{\text{ads}}}} \to {\text{dye}}\;{\text{degradation}} $$

## Conclusions

In conclusion, different nanoparticles of Ag–ZnO NPs were successfully synthesized through environmental friendly novel green method using potato waste. XRD and FTIR studies reveal that Ag–ZnO nanocomposites have highly crystalline nature, good chemical characteristics and exhibit perfect morphological entity. The as-synthesized 2% Ag–ZnO nanocomposites possess maximum catalytic activity (~ 96%) to degrade the MB dye within 80 min by visible light, which demonstrates the potential application towards the wastewater purification with environmental friendly material. This enhanced photocatalytic performance of 2% Ag–ZnO nanocomposites was mainly ascribed to the decreased recombination rate of photogenerated charge carriers stimulated by the anchoring of AgNPs on the surface of ZnO. These photocatalysts can be applied as fruitful photocatalytic material on industrial scale for the exploitation of structurally stable and complex dyestuff. This study offers a green and non-toxic method containing biomaterials to synthesize various nanomaterials using this eco-friendly preparation route which could be extend to use other natural sources including rice, corn and other grains.
